# Targeted therapy for knee osteoarthritis: From basic to clinics

**DOI:** 10.1097/MD.0000000000043686

**Published:** 2025-08-15

**Authors:** Li Chen, Feng-Lan Huang, Qi Tang, Zhi-Kai Zhao, Zhen-Yan Ye, Juan-Hong Liang

**Affiliations:** a West China School of Public Health and West China Fourth Hospital, Sichuan University, Chengdu, Sichuan, China; b The University of Sydney, NSW, Sydney, Australia; c School of Clinical Medical, Chengdu Medical College, Chengdu, People’s Republic of China.

**Keywords:** clinical trials, inhibitors, knee osteoarthritis, nanocarriers, targeted therapy

## Abstract

As the aging population grows and lifestyle factors become more prevalent, the incidence of knee osteoarthritis (KOA) is expected to continue to increase in the coming decades. This presents a substantial public health challenge with an impact on the quality of life of the affected individuals. The absence of targeted therapeutic interventions tailored specifically for KOA underscores the recognition of this condition as a significant medical concern characterized by an urgent unmet need for effective treatments. Despite advances in understanding its pathophysiology and progression, there remains a gap in the availability of therapies capable of adequately addressing the diverse clinical manifestations and underlying mechanisms of KOA. Fortunately, numerous novel targeted therapies, including biological, nanotechnology, gene, and cell therapies, are currently undergoing clinical trials for KOA treatment. Advancements in drug nanocarriers and delivery systems have demonstrated the potential to enhance the efficacy of therapeutic agents for KOA. In this review, we summarize all the advancements in targeted therapy for KOA, including small-molecule inhibitors, monoclonal antibodies, nanocarrier-based therapy, gene therapy and cytotherapy. By analyzing the latest breakthroughs in pharmaceutical therapies and relevant clinical data, this review serves as a valuable resource for clinicians and researchers involved in the ongoing quest for effective KOA treatments and provides hopes for improved management and outcomes for patients with this debilitating condition.

## 1. Introduction

**Figure 1.**
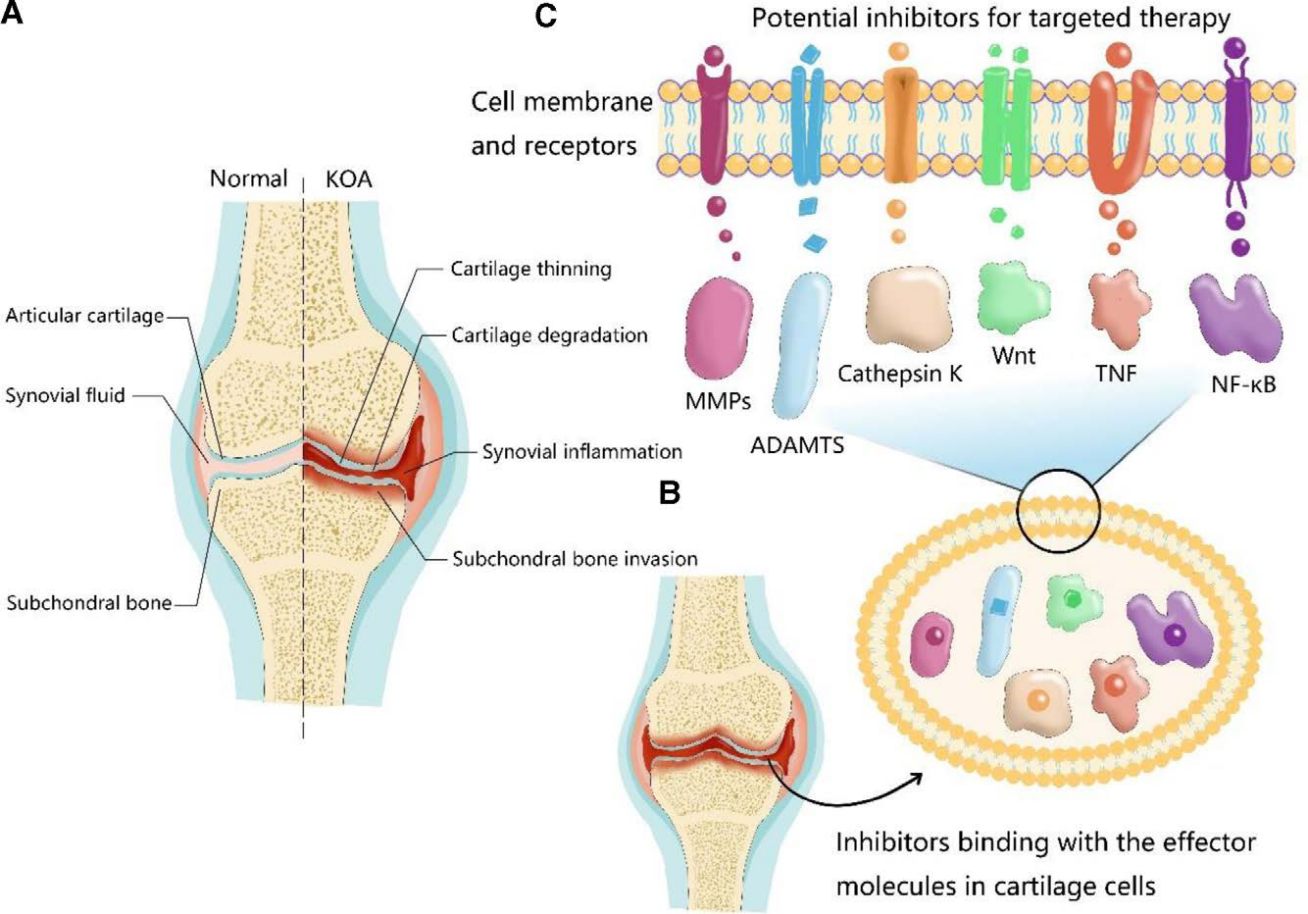
Phenotypes of KOA (A) and inhibitors for KOA therapy binding with the effector molecules in cartilage cells (B) to regulate the expression of proteins and genes (C). KOA = knee osteoarthritis.

Osteoarthritis (OA) is recognized as the predominant degenerative arthropathy, characterized by intricate dysregulation of the entire synovial joint, encompassing structural aberrations in hyaline articular cartilage, depletion of integral subchondral bone, tissue hypertrophy, heightened vascularity in the synovium, and instability in tendons and ligaments, affecting a global population exceeding 500 million individuals.^[1,2]^ Knee osteoarthritis (KOA), the most common type of OA, constitutes approximately 85% of the total prevalence of OA cases worldwide, thereby presenting a substantial public health challenge anticipated to persist in the forthcoming decades.^[3,4]^ KOA exerts its influence not only on the cartilage but also on the entirety of the joint structure^[5]^ (Fig. 1A). The central characteristic of KOA is the degradation of articular cartilage, in which an imbalanced biomechanical microenvironment and diverse biological factors disrupt cartilage homeostasis, leading to degradation of the extracellular matrix (ECM) enriched in collagen and proteoglycans, as well as articular surface fibrosis, cell death, vascular invasion, and so on.^[6]^ This progressive destruction stimulates chondrocytes to augment anabolism through compensatory hypertrophy, resulting in the concurrent generation of matrix degradation products and pro-inflammatory mediators, thereby expediting the development of KOA. In addition, the involvement of the tissues in and around the joint is frequently followed by the KOA progression.^[7]^ These multifactorial changes collectively worsen the clinical symptoms of KOA and complicate its management.

Over an extended period, significant efforts and resources have been dedicated to the development of therapies aimed at enhancing care, quality of life, and pain relief in individuals with KOA. Non-pharmaceutical approaches, which include patient education, exercise, and weight management, constitute fundamental elements in the management of KOA.^[8,9]^ Pharmaceutical strategies have primarily focused on alleviating symptoms and attempting to impede or halt the underlying biological processes that contribute to tissue damage. Despite numerous studies, a conclusive treatment for KOA remains elusive, and there are currently no medications to halt the progression of KOA. Current guidelines derive recommendations for diverse OA treatment modalities from medical literature. Nonsteroidal anti-inflammatory drugs (NSAIDs), analgesics, and intra-articular corticosteroid injections show limited efficacy in the extended duration, whereas the use of opioids may lead to adverse outcomes.^[10–12]^ In recent years, innovative treatments have emerged owing to enhanced insights into KOA and the rapid development in nanotechnology and drug delivery system. Moreover, scientists and clinicians have invested substantial efforts in elucidating pivotal molecules, signaling pathways, biological processes and the suitable time of the intervention, which holds promise as a potential therapeutic targets to attenuate or limit damage to synovial joints.^[2,13]^

In this review, we perform a comprehensive search of English-language articles using databases such as PubMed, Google Scholar, and the NIH, containing the following terms: “knee osteoarthritis,” “targeted therapy,” “‘clinical trials,’” “inhibitors,” and “nanocarriers” and explore all the advancements in targeted therapy for KOA, as well as the relevant clinical data, elucidating the efficacy and safety profiles of these novel therapeutic approaches in clinical settings. Additionally, we engage in a comprehensive discussion regarding the main challenges that need to be addressed for the continued advancement and refinement of these targeted therapeutic strategies, aiming to provide a deep understanding of the current landscape of KOA therapy and offer valuable insights for future research and clinical practice in the field.

## 2. Management of KOA

### 2.1. Pharmaceutical approaches

Clinically pharmaceutical approaches to KOA have primarily concentrated on alleviating symptoms or modifying the disease by topical, oral, or injectable administration (Table 1). First-line medications, such as NSAIDs, analgesics, and glucocorticoids, have been shown to be effective for symptom control.^[14,15]^ However, there are notable cardiovascular, gastrointestinal, and renal risks associated with the oral administration of NSAIDs. Concerns have also arisen regarding the effectiveness and safety of opioid analgesics in oral therapy. Consistent daily use of opioid analgesics can lead to a degree of tolerance and physical dependence.^[16]^ It is essential to acknowledge that acetaminophen is no longer the first-line recommended analgesic in guidelines, primarily because of its effectiveness and the presence of various unwanted side effects.^[17,18]^ An additional category of commonly employed medications falls under the designation of cartilage protectors, encompassing products containing substances like glucosamine,^[19,20]^ chondroitin sulfate^[20]^ and collagen hydrolysates.^[21]^ Despite of the fact that many studies have suggested anti-inflammatory and analgesic effects of these cartilage protectors in KOA, leading to relief from clinical symptoms and a deceleration in disease progression, the observed effects are only marginally better than those of a placebo. Limited favorable evidence and relevance support these findings.^[22,23]^ Therefore, some clinical guidelines have issued either negative or weak recommendations for all glucosamine and chondroitin products, including those of pharmaceutical grade.^[17]^ Several innovative targeted therapies for KOA have been developed to enhance drug delivery specificity and efficacy. We will discuss these in detail in the following “Targeted therapy strategies” section.

**Table 1:** Management and mechanisms of KOA.

Management of KOA			Mechanisms of KOA	
Pharmaceutical approaches	Non-pharmaceutical approaches	Surgical management	Pathogenesis	Molecular mechanisms of KOA
NSAIDS	Health education	OAK	Excessive proliferation of chondrocytes	MMPs, ADAMTS↑
Analgesics	Regular exercise including aerobic, strengthening, and resistance exercises	UKA	Abundance of ECM	IL-1β, IL-6, TNF-α↑
Glucocorticoids	Weight loss	TKA	Synovial inflammation	NF-κB, MAPK, PI3K/Akt signaling pathway
Targeted therapy strategies	Physical therapy including therapeutic ultrasound, electrical stimulation, phototherapy, hydrotherapy, magnet therapy, acupuncture and moxibustion	–	Breakdown of cartilage tissue	–

ADAMTS = a disintegrin and metalloproteinase with thrombospondin motifs, ECM = extracellular matrix, IL-1β = interleukin-1, IL-6 = interleukin-6, KOA = knee osteoarthritis, MMPs = matrix metalloproteinases, NSAIDs = nonsteroidal anti-inflammatory drugs, OAK = osteotomy around the knee, TKA = total knee arthroplasty, TNF-α = tumor necrosis factor-alpha, UKA = uknee arthroplasty.

### 2.2. Non-pharmaceutical approaches

Virtually all guidelines endorse the incorporation of health education, regular exercise and weight loss throughout the distinct pathological stages of KOA (Table 1), which should precede the initiation of first-line treatment.^[17]^ The recommendations from the guidelines support the inclusion of aerobic, strengthening, and resistance exercises.^[24]^ Nevertheless, the impact of exercise intensity on the outcomes of KOA rehabilitation, particularly in the acute stage, remains unclear. Improper exercise prescription may exacerbate KOA.^[25]^ Weight loss contributes to a decrease in the pressure on the knee joint, leading to improvements in physical function and biomechanics when combined with exercise. Additionally, physical therapy has shown a significant therapeutic effect on KOA through modalities such as therapeutic ultrasound, electrical stimulation, phototherapy, hydrotherapy, and magnet therapy, which can effectively alleviate symptoms.^[26]^ This renders it suitable for emergency management during the acute phase of KOA.^[27]^ Acupuncture and moxibustion as physical therapies in traditional Chinese medicine also play specific roles in pain relief and functional restoration in the treatment of KOA.^[28]^

### 2.3. Surgical management

Osteotomy around the knee as a conservative treatment offers value in the surgical management of KOA in younger patients^[29]^ (Table 1). This approach facilitates significant improvements in pain reduction and functional outcomes without the need for irreversible arthroplasty.^[30]^ The preservation of the joint’s anatomical structure through osteotomy offers advantages such as maintaining proprioception and facilitating swift recovery of joint functional efficacy. This, in turn, significantly slows the progression of KOA. Knee arthroplasty is a nonconservative surgical management for severe KOA and is regarded as a secure, widely accepted, and cost-effective therapeutic intervention, offering enduring benefits.^[30]^ Unquestionably, unicompartmental knee arthroplasty has evolved as the established surgical strategy for individuals with total cartilage loss limited to a single femorotibial compartment.^[31,32]^ Upon satisfying the appropriate criteria and adhering to surgical principles, full knee functionality can be restored using unicompartmental knee arthroplasty. In cases of severe arthritis, consideration for total knee arthroplasty is warranted, contingent upon the failure of nonoperative interventions.^[33]^

## 3. Molecular mechanisms of KOA

**Figure 2.**
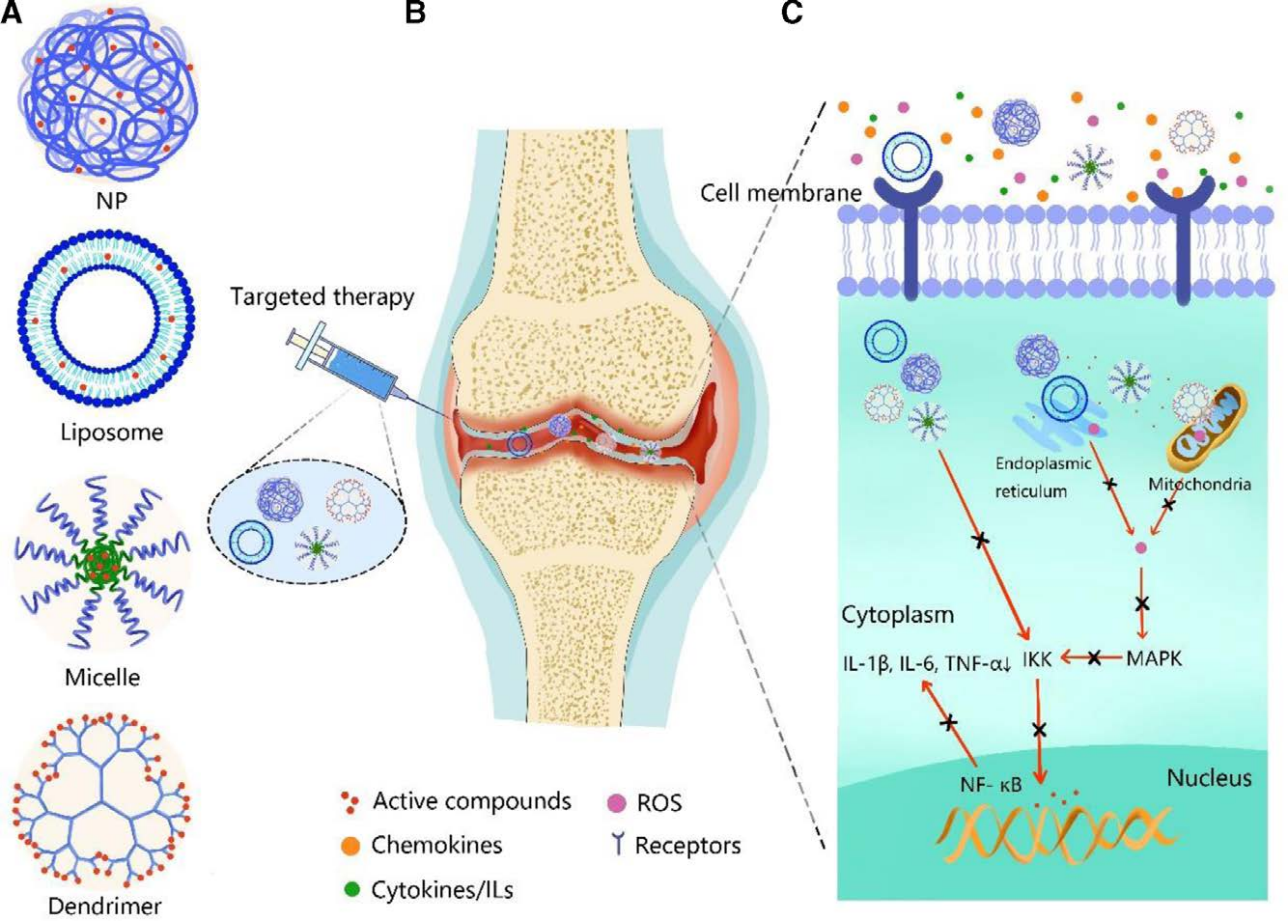
Schematic of nanocarrier-based therapy. (A) the various nanocarriers loading active compounds, (B) intra-articular administration for targeted therapy of KOA, (C) the therapeutic effects are obtained by the inhibition of NF-κB and MAPK signaling pathway. KOA = knee osteoarthritis.

KOA is primarily characterized by the excessive proliferation of chondrocytes, which are specialized cells within cartilage tissue, coupled with a gradual decline in the abundance of ECM proteins, leading to the breakdown of cartilage tissue, contributing to joint degeneration and dysfunction (Table 1). Among the aberrant molecular mechanisms of KOA, there is a notable upregulation in the synthesis of degrading proteases, including matrix metalloproteinases (MMPs) and a disintegrin and metalloproteinase with thrombospondin motifs (ADAMTS) (Fig. 1C). Specifically, these enzymes target type II collagen, a key structural component of cartilage that leads to cartilage degradation.^[34]^ Interleukin-1 (IL-1β), which is primarily secreted by chondrocytes, osteoblasts, synovial cells, and leukocytes, is involved in the pathogenesis of KOA. IL-1β triggers downstream signaling cascades involving NF-κB and MAPK pathways by binding to its receptor on synoviocytes (Fig. 2C), and then induces the transcription of genes encoding inflammatory mediators, including MMPs and cyclooxygenase-2 (COX-2), which are responsible for cartilage degradation.^[35,36]^ Similarly, Interleukin-6 (IL-6) collectively accelerates KOA progression by stimulating MMP production, promoting cartilage permeability, and enhancing osteoclast formation.^[37]^ Additionally, upon binding to its receptors, tumor necrosis factor-alpha (TNF-α) can activates the NF-κB and PI3K/Akt signaling pathways to initiate a cascade of events that culminate in the production of pro-inflammatory mediators.^[38]^ These findings highlight the possible molecular mechanisms mediating various aspects of KOA pathophysiology and underscore its potential as a therapeutic target for disease management.

## 4. Targeted therapy strategies

Several innovative approaches have been devised to enhance drug delivery specificity and efficacy in joint tissues for KOA according to aberrant molecular mechanisms. Active and passive targeting techniques have been tailored to target specific cells and tissues within the joint, such as cartilage, synovium/synoviocytes, chondrocytes, and the joint microenvironment, including the synovial fluid^[39]^ (Fig. 1B). These targeted therapeutic strategies aim to improve drug retention and concentration at the joint by leveraging the unique characteristics and physiological properties of joint tissues, such as cell surface markers or tissue-specific receptors, thereby enhancing therapeutic outcomes while minimizing off-target effects. Herein, we summarize the recent advancements in targeted therapy for KOA, with a focus on a diverse array of therapeutic approaches, including small-molecule inhibitors, monoclonal antibodies, nanocarrier-based therapy, gene therapy and cytotherapy.

### 4.1. Small-molecule inhibitors

Small-molecule inhibitors, characterized by their molecular weight of <1000 Da, constitute a fundamental class of organic compounds widely utilized in biomedical research and targeted drug development.^[40]^ Small-molecule inhibitors offer a versatile approach for manipulating cellular processes and influencing disease pathways.^[41]^ Their relatively small size allows them to penetrate cellular membranes efficiently, enabling them to interact with intracellular targets and exert precise regulatory effects on biological pathways.^[42]^ Notably, the inhibitors exhibited promising inhibitory effects on KOA progression, suggesting their utility in mitigating the disease’s pathological processes and offering potential avenues for therapeutic intervention in KOA.^[43,44]^ Among them, MMPs inhibitors, ADAMTS inhibitors, Cathepsin K inhibitor and Wingless-type MMTV integration site family (Wnt) signaling inhibitors have been well evaluated, offering potential therapeutic benefits for KOA patients.

#### 4.1.1. MMPs inhibitors

MMPs are a family of enzymes implicated in KOA owing to their involvement in cartilage degradation.^[45,46]^ The specific mechanism involves MMPs breaking down the extracellular matrix components of cartilage, contributing to its deterioration in KOA. Consequently, the development of synthetic small-molecule inhibitors targeting MMPs holds promise as a potential therapeutic strategy for KOA (Fig. 1C). By inhibiting MMP activity, these molecules aim to mitigate cartilage degradation and thereby alleviate KOA symptoms, thereby offering a potential route for disease management and treatment. As recently reported, the allosteric MMP-13 inhibitor, AQU-019 is a promising candidate that has been optimized to enhance its potency, metabolic stability, and oral bioavailability.^[47]^ However, available preclinical data remains insufficient to support their commercialization.

#### 4.1.2. ADAMTS inhibitors

ADAMTS is also known to play crucial roles in the degradation of cartilage matrix components, contributing to the progression of KOA.^[48]^ Consequently, the development of small-molecule inhibitors targeting ADAMTS has emerged as a logical therapeutic approach for mitigating cartilage degradation and attenuating KOA progression (Fig. 1C).^[49]^ Inhibition of ADAMTS presents a potentially safer alternative to targeting MMP in the context of KOA.^[50,51]^ GLPG1972/S201086, identified as a small-molecule inhibitor targeting ADAMTS5, exhibits promising chondroprotective properties in preclinical models of OA.^[52,53]^ In phase I clinical trials involving healthy individuals (NCT03311009, https://clinicaltrials.gov/), GLPG1972/S201086 demonstrated a favorable safety profile, indicating its tolerability.^[54,55]^ In a global phase II clinical trials, significant reductions in cartilage thickness of patients for 52 weeks were observed, indicating that GLPG1972/S201086 can be considered a notable structural advantage of a disease-modifying drug candidate for KOA (NCT03595618). However, the study did not achieve its primary and secondary endpoints, and no dose-response relationship was observed.^[56]^ The treatment was generally well-tolerated, but the lack of efficacy led to the discontinuation of OA development. While GLPG1972/S201086 did not demonstrate efficacy as a monotherapy for OA, its potential synergies with other therapeutic agents have not been fully explored. Combining it with other treatments could theoretically enhance therapeutic outcomes, however, further research is necessary to evaluate such combinations to explore their potential for clinical application.

#### 4.1.3. Cathepsin K inhibitor

Cathepsin K, a cysteine protease, exhibits predominant expression within osteoclasts, playing a pivotal role in bone resorption by facilitating the degradation of bone matrix proteins (Fig. 1C).^[57,58]^ In preclinical studies, MIV-711 a cathepsin K inhibitor, was shown to reduce cartilage damage and preventing bone resorption in animal models.^[59]^ Moreover, it has been shown to decrease biomarkers associated with bone and cartilage remodeling, suggesting its potential as a protease inhibitor for the management of KOA progression. In a phase II clinical trial involving individuals with symptomatic KOA, MIV-711 treatment demonstrated notable outcomes (NCT02705625). MIV-711 was generally well-tolerated over both the initial 26-week period and subsequent 6-month extension. Serious adverse events were infrequent and none were deemed treatment-related. Specifically, patients receiving MIV-711 exhibited reduced bone remodeling, as evidenced by magnetic resonance imaging assessments indicating decreased bone area, compared to those receiving a placebo. Moreover, over the 26-week study period, individuals treated with MIV-711 experienced less cartilage loss than those in the placebo group.^[60]^ A primary limitation of these studies was their relatively short duration, which may have been insufficient to observe symptom benefits following structural modification. Additionally, the lack of statistically significant pain reduction despite structural improvements suggests that longer treatment periods or combination therapies may be necessary to achieve symptomatic relief. Given the ability of MIV-711 to modify the joint structure, combining it with agents that provide symptomatic relief could offer a comprehensive treatment approach for OA. Further research is warranted to explore such combination therapies and determine whether extended treatment durations with MIV-711 alone can yield significant symptom improvement in larger-scale clinical trials.

#### 4.1.4. Wnt signaling inhibitors

Wnt signaling pathways, primarily mediated by β-catenin and involving a myriad of proteins, play multifaceted roles in various physiological processes not only within joints but also in other tissues throughout the body.^[61]^ The widespread involvement of Wnt signaling complicates the selective targeting of this pathway for the treatment of KOA. By modulating Wnt-related proteins, interventions may mitigate the aberrant signaling cascades implicated in KOA development and progression.^[62,63]^ Lorecivivint (LOR), an inhibitor of the Wnt signaling pathway, was discovered using high-throughput screening methods. Preclinical studies have shown that the inhibitory effects of LOR on the Wnt signaling pathway contribute to its anti-inflammatory and chondroprotective properties. Interestingly, these effects appear to be independent of β-catenin and instead involve the inhibition of 2 intranuclear kinases: cdc2-like kinase (CLK2) and tyrosine phosphorylation-regulated kinase (DYRK1A).^[64]^ Moreover, phase I-III clinical trials for LOR have been completed (NCT02536833, NCT03122860, NCT04385303, NCT03928184, NCT05603754) and the results showed that the administration of LOR via intra-articular injection had a favorable safety profile, without indications of systemic drug exposure. Additionally, LOR led to improvements in KOA symptoms, including pain reduction and enhanced joint function, compared with placebo. Moreover, imaging analyses have suggested potential disease-modifying effects, such as reduced cartilage degradation.^[65–68]^ While the results are promising, the study duration was limited to 52 weeks, which may not capture long-term efficacy and safety outcomes. Further studies are necessary to confirm the sustained benefits and monitor any potential long-term adverse effects.

#### 4.1.5. Others

In addition to MMPs, ADAMTS, Cathepsin K inhibitors, Wnt signaling inhibitors, and various other small-molecule inhibitors, including IL-6, TNF and NF-κB signaling pathways, have demonstrated potential in inhibiting the onset and progression of KOA. Recently, a small-molecule compound, SC75741, was reported to exhibit protective effects against articular joint destruction in preclinical studies by inhibiting key inflammatory mediators, including NF-κB, TNF-α, and IL-6. By targeting the NF-κB signaling pathway, SC75741 exhibited therapeutic potential as a small-molecule inhibitor capable of attenuating miR-21/NF-κB-driven KOA progression.^[69,70]^ Further investigation of its mechanism of action and safety profile is warranted to validate its clinical utility in KOA management. Indeed, by employing advanced screening methodologies, such as combinatorial chemistry and computational modeling techniques, researchers have identified promising small-molecule inhibitors that exhibit inhibitory activity against key enzymes or signaling pathways implicated in KOA progression. These findings highlight the potential of high-throughput screening as a valuable approach for discovering novel therapeutics for KOA and advancing our understanding of the molecular mechanisms underlying the disease.^[71–73]^

### 4.2. Monoclonal antibodies

Monoclonal antibodies (mAbs) have also been developed as versatile therapeutic agents for modulating cellular processes and impacting KOA pathology. They selective target specific proteins or interfer with crucial biochemical reactions, including ADAMTS,^[51,74,75]^ Interleukins (ILs),^[76,77]^ TNF,^[78]^ nerve growth factor (NGF),^[79]^ and CC-chemokine ligand 17 (CCL17).^[80,81]^ MAbs offer a precise and potent means of modulating disease pathways. Through their ability to bind with high affinity to specific antigens, mAbs can block key signaling pathways involved in KOA pathogenesis, thereby attenuating inflammation, cartilage degradation, and joint damage. Their specificity and targeted action make them promising candidates for the development of novel KOA treatments with potentially fewer off-target effects than small-molecule inhibitors. Many clinical trials have been performed for the evaluation of mAbs, including NCT03583346 for neutralizing antibodies against ADAMTS, NCT03304379 and NCT02528188 for Fasinumab and Tanezumab (neutralizing antibodies against NGF), and NCT03485365 for anti-CCL17 antibodies. However, no mAbs have been successfully approved for use as alternative clinical therapies for KOA. Tanezumab, the most promising neutralizing antibody, was announced that a Phase III study showed the 10 mg dose of tanezumab significantly improved chronic low back pain at 16 weeks compared to placebo (NCT02528253). Some Phase III trials have reported that tanezumab effectively reduced hip pain (NCT00863304) and pain associated with KOA (NCT02528188). However, its use raised concerns regarding joint-related adverse events, leading to the denial of its application by the Food and Drug Administration (FDA) during phase III studies.^[82]^ Similarly, Fasinumab, another monoclonal antibody targeting NGF, was developed to manage pain associated with OA. A clinical trial identified by NCT03304379 evaluated its efficacy and safety in patients with moderate-to-severe KOA. However, Fasinumab faces challenges and uncertainties regarding its safety profile and clinical outcomes, posing dilemmas for further development and regulatory approval. Therefore, the primary limitation of Tanezumab and Fasinumab lies in their safety profile, particularly the risk of joint-related adverse events, which has led to the suspension of certain clinical trials and regulatory hesitancy. Additionally, the variability in efficacy between different dosages, suggests that the optimal dosing requires further investigation. In addition, as with any monoclonal antibody therapy, there is a possibility of immunogenicity, which could affect the long-term efficacy and safety.

### 4.3. Nanocarrier-based therapy

#### 4.3.1. Nanoparticles

Nanoparticles (NPs) are frequently employed in studies focusing on targeted therapy of KOA by enhancing drug penetration across the cartilage matrix regulating drug pharmacokinetics, improving efficacy, and reducing the toxicity of therapeutic agents (Fig. 2A and B).^[83–85]^ Through the manipulation of physicochemical properties or surface modifications with specific moieties, NPs can be engineered with functional groups to selectively target components or cells in KOA therapy. Natural polymers, such as chitosan,^[86,87]^ silk fibroin,^[88,89]^ albumin,^[90,91]^ and chemically synthesized nanomaterials, such as poly (lactic-co-glycolic) acid (PLGA),^[92,93]^ polylactic acid (PLA),^[94]^ polyurethane,^[95]^ and different polymer combinations,^[96]^ are the most frequently used to prepare NPs for drug delivery in KOA therapy. In addition to chemically polymeric nanoparticles, organometallic or inorganic materials have also been utilized to form nanoparticles for targeted drug delivery in KOA therapy. Hollow mesoporous silica nanoparticles capped with chitosan to construct pH-responsive nanoparticle are recognized as promising entities in the field of KOA therapy.^[97]^

Recently, there has been a notable increase in the development of multifunctional NPs for KOA therapy to amplify the targeting profile and reduce side effects, including microenvironment sensitive NPs,^[98,99]^ ligand-modified NPs,^[100]^ antibody-modified NPs,^[101]^ magnetic NPs,^[102]^ and bionic NPs.^[103]^ Among these, the most prevalent and effective strategy is cartilage targeting, which involves the modification of NPs with specific functional groups or ligands that possess an inherent affinity for cartilage tissue. By tailoring the surface properties of NPs through the incorporation of cartilage-targeting moieties, such as peptides or antibodies, researchers aim to enhance the specificity and selectivity of drug delivery to affected joint tissues. As reported in the literature, drug delivery systems based on NPs hold potential for KOA therapy, offering benefits such as specific drug distribution, prolonged drug release, and enhanced drug retention. To date, however, there have been no reports on the use of NPs as drug carriers in clinical trials for KOA. The complex preparation procedures associated with NPs and potential toxicity concerns may hinder their clinical application for KOA treatment.

#### 4.3.2. Liposomes

Liposomes possess a unique bilayer membrane, where the hydrophilic head groups face the aqueous environment, whereas the lipophilic tails are sequestered within the interior (Fig. 2A).^[104]^ This amphiphilic nature enables liposomes to efficiently encapsulate both hydrophilic and lipophilic molecules within their aqueous core and lipid bilayer, respectively.^[105]^ Beyond their structural elegance, liposomes have earned distinction as premier nano-drug carriers, securing approval from the stringent regulatory standards set forth by the FDA.^[106]^ Recent studies have focused on incorporating active, small-molecule compounds such as rapamycin and lornoxicam, which typically have low bioavailability, into liposomal formulations for the treatment of KOA. These findings have demonstrated enhanced therapeutic efficacy and improved bioavailability compared with conventional formulations.^[107,108]^ Similarly, surface modifications of liposomes with functional targeting groups is a prevalent approach in drug delivery strategies. By capitalizing on the abundant presence of type II collagen in cartilage, researchers have explored the modification of liposomes with type II collagen antibodies to achieve cartilage-specific targeting for drug delivery.^[109]^ Studies have reported promising outcomes, suggesting that these tailored liposomes can effectively be accumulated into cartilage tissues, thereby enhancing the precision and efficacy of drug delivery to target sites within the joint. Other advanced strategies have been applied to liposomes.^[110,111]^ This targeted approach holds significant potential for optimizing therapeutic outcomes while minimizing off-target effects, thereby advancing the field of precision medicine for KOA treatment.

In clinical trials, liposomal bupivacaine has attracted much attentions and positive results have been obtained. In this review we summarized the targeted therapy strategies being investigated in KOA clinical trials (Table 2). A double-blinded, randomized controlled trial (NCT04910165) was perform recently, which contrasted the use of liposomal bupivacaine with ropivacaine as preoperative interventions before TKAs. Liposomal bupivacaine in peripheral regional nerve blocks is associated with reductions in pain intensity, shorter hospital stays, decreased use of opioids among inpatients, enhanced WOMAC scores, and good safety.^[112]^ Additionally, a phase III, randomized, double-blind, placebo- and active-controlled study was conducted to evaluate the efficacy and safety of liposomal formulation of dexamethasone (TLC599) in patients with KOA (NCT04123561). TLC599 was designed to provide prolonged pain relief and improved joint function in patients with KOA through sustained delivery of dexamethasone, with the aim of minimizing systemic exposure and associated risks. Adverse events, vital signs, and laboratory parameters were monitored to assess the safety profile of TLC599. The results showed that TLC599 provided prolonged pain relief and improved joint function in KOA patients through the sustained delivery of dexamethasone. Compared with low-dose dexamethasone (4mg) administration and placebo, the incidence rate of adverse events did not increase after the administration of TLC599 with a dose of 12mg dexamethasone. As pioneers in the realm of nanomedicine for KOA, liposomes continue to fuel innovation and inspire the development of novel drug delivery systems aimed at address the complex challenges of modern pharmacotherapy.

**Table 2:** Targeted therapy strategies being investigated in KOA clinical trials.

Targeted therapy strategies	Type of drug/carrier	Drug name	Administration routes	Safety and	Effectiveness	Clinicaltrials.gov identifier	Current phase of development	References of clinical data
Small-molecule inhibitors	ADAMTS5 inhibitor	GLPG1972/S201086	Oral	Well-tolerated	Inefficacy	NCT03311009, NCT03595618	Phase II	[54–56]
	Cathepsin K inhibitor	MIV-711	Oral	Well-tolerated	Significant reduction of the progression of bone area and cartilage thinning	NCT02705625	Phase II	[60]
	Wnt signaling inhibitors	Lorecivivint	Intra-articular	Well-tolerated	Pain reduction and enhanced joint function	NCT02536833, NCT03122860, NCT04385303, NCT03928184, NCT05603754	Phase III	[65–68]
Monoclonal antibodies	Neutralizing antibody against ADAMTS5	M6495	Subcutaneous	Well-tolerated	Effective inhibition of aggrecan degradation	NCT03583346	Phase II	/
	Neutralizing antibody against NGF	Tanezumab, Fasinumab	Subcutaneous	The potential for joint-related adverse events	Significant pain relief	NCT03304379, NCT02528188	Phase III	[82]
	Anti-CCL17 antibody	GSK3858279	Intravenous, subcutaneous	Favorable safety and tolerability	Significant improvements in knee pain	NCT03485365	Phase I	/
Nanocarrier-based therapy	Liposomes	Bupivacaine	Intra-articular	Good safety	The prolonged analgesia	NCT04910165	Phase III	[112]
	Liposomes	Dexamethasone	Intra-articular	Minimization of systemic exposure and associated risks	The prolonged pain relief and improved joint function	NCT04123561	Phase III	/
Gene therapy	Chondrocytes	Tissuegene-C	Intra-articular	Well-tolerated	Increase of cartilage thickness and slower rates of subchondral bone surface area growth	NCT02072070	Phase III	[113]
	Nonviral gene therapy	XT-150	Intra-articular	Well-tolerated	Significant improvements in pain and function	NCT04124042	Phase II	/
	Adeno-associated viral vectors	Sc-raav2.5IL-1Ra	Intra-articular	–	–	NCT02790723	Phase I	/
	Adeno-associated viral vectors	GNSC-001	Intra-articular	–	–	NCT05835895	Phase I	/
	Adeno-associated viral vectors	FX201	Intra-articular	–	–	NCT04119687	Phase I	/
Cytotherapy	Mesenchymal stem cells	AT-mscs	Intra-articular	Joint-related adverse events	Limited pain relief and functional improvement	NCT01183728, NCT03869229, NCT05081921, NCT05783154	Phase II	[114–117]
	MSC-exos	Exooa-1	Intra-articular	–	–	NCT05060107	Phase I	/

ADAMTS = a disintegrin and metalloproteinase with thrombospondin motifs, KOA = knee osteoarthritis, MSC-Exos = mesenchymal stem cell-derived exosomes.

“–” represents no results posted; “/” represents no reference available.

#### 4.3.3. Other

Micelles with diameters typically ranging between 5 and 100 nm arise through the spontaneous arrangement of amphiphilic polymers within water-based solutions,^[118,119]^ making them suitable carriers for a wide range of therapeutic agents in nanomedicine applications (Fig. 2A and B).^[120]^ The acidic microenvironment and elevated levels of matrix metalloproteinase-13 (MMP-13) serve as characteristic biomarkers associated with KOA, providing valuable targets for the development of micelle-based drug delivery systems.^[121]^ Among these systems, MMP-13 responsive and pH sensitive polymer (MR-PPL) micelles, poly (β-amino ester) micelles, have emerged as promising platforms for KOA treatment.^[121–123]^ These micelles can respond to the acidic conditions prevalent in KOA-affected joints, facilitating targeted drug release with heightened specificity and efficacy. Using these OA markers, researchers aim to enhance the therapeutic outcomes of micellar drug delivery, offering a promising direction for precision medicine in KOA management.

Dendrimers comprising 3 distinct components – core, branches, and shell – exhibit a well-defined architecture that lends itself to precise functionalization and manipulation (Fig. 2A and B).^[124,125]^ The outer shell of dendrimers serves as a versatile platform for the attachment of cargo molecules or targeting ligands, allowing tailored modifications to enhance their therapeutic or diagnostic capabilities.^[126,127]^ Through conjugation strategies, dendrimers can be engineered to selectively deliver payloads to specific cellular targets or tissues, offering immense potential for drug delivery applications.^[128]^ A novel dendrimer approach involving kartogenin (KGN), polyamidoamine (PAMAM), and polyethylene glycol (PEG) has been reported and 2 distinct conjugates, PEG-PAMAM-KGN and KGN-PEG-PAMAM, were synthesized. Through this strategy, they enhanced the release profile of KGN and amplified the chondrogenic effects within KOA joints.^[129]^ The conjugation of insulin-like growth factor 1 with PAMAM and PEG not only facilitated controlled release kinetics but also potentiated the therapeutic efficacy of the drug, suggesting a promising avenue for the development of targeted and sustained drug delivery systems for KOA treatment.^[130]^

Despite the growing interest in nanomedicine for KOA treatment, the clinical application of micelles or dendrimers remains relatively limited. This restraint may stem from inherent drawbacks associated with micellar or dendrimer systems, such as their inefficacy in encapsulating hydrophilic drugs, the uncontrolled burst release effect, and concerns regarding potential toxicity. These limitations underscore the need for continued research and innovation to overcome challenges associated with nanocarrier-based therapies for KOA. Addressing these concerns could unlock the full potential of micelles and dendrimers in delivering therapeutics for KOA management, paving the way for more effective and safer treatment options.

### 4.4. Gene therapy

In the context of KOA treatment, gene therapy does not primarily aim to restore aberrant KOA-related genes to their normal states. Instead, it serves as a delivery platform designed to inhibit the expression of genes implicated in KOA progression or to induce the overexpression of therapeutic factors to mitigate KOA pathology.^[131]^ This approach involves targeted modulation of gene expression within affected joint tissues, with the ultimate goal of alleviating symptoms and halting disease progression. By manipulating gene expression profiles, the gene therapy holds promise for providing long-term therapeutic benefits and potentially altering the underlying mechanisms driving KOA pathogenesis.^[132]^

The clinical effectiveness of TissueGene-C (TG-C) as a cell and gene therapy designed for KOA treatment was evaluated in a multicenter, double-blind, phase III clinical trial (NCT02072070).^[113,133]^ Patients receiving TG-C treatment displayed increased cartilage thickness, slower rates of subchondral bone surface area growth in various knee regions and improved serum index levels. These findings suggest potential of TG-C as a disease-modifying drug for KOA treatment. TG-C is generally well-tolerated in clinical studies. The most commonly reported adverse events were mild to moderate in severity, including localized joint pain and swelling. No serious adverse events related to the treatment have been reported. Other clinical trials of gene therapy for KOA are available at https://clinicaltrials.gov/ (NCT04124042, NCT02790723, NCT05835895, and NCT04119687). XT-150 is an investigational nonviral gene therapy designed to deliver IL-10v, a modified variant of the anti-inflammatory cytokine interleukin-10, aimed at treating moderate-to-severe KOA in NCT04124042. Although the primary endpoint, – achieving at least 30% improvement in WOMAC pain score by day 180, – showed no statistically significant difference between the XT-150 and placebo groups, post hoc analyses in stage B indicated that participants receiving 2 doses of 0.45 mg XT-150 experienced significant improvements in pain and function by day 360 compared to those receiving a single dose. The primary endpoint of the study was not met, suggesting the need for further research to optimize dosing regimens and confirm efficacy. Additionally, the improvements observed in post hoc analyses require validation in larger, randomized trials. A recent clinical study on adeno-associated virus gene therapy, including Sc-rAAV2.5IL-1Ra, GNSC-001 and FX201 for KOA by intra-articular injection was also conducted (https://clinicaltrials.gov/, NCT05835895). As these are ongoing clinical trials, comprehensive data on efficacy and safety are not yet available.

Therefore, the advancement of gene therapy in KOA is rapidly progressing and fueled by continuous research and technological innovations. Additionally, advancements in gene delivery systems, such as viral vectors and nanoparticles, have facilitated the effective and targeted delivery of therapeutic genes to KOA-affected tissues. Therefore, the development of gene therapy holds promise for revolutionizing KOA treatment in the future. However, the research and development of gene therapies involve substantial investments, often exceeding 100s of millions of dollars and producing viral vectors or other gene delivery systems requires highly specialized facilities and stringent quality control, contributing to high production costs, resulting in many approved gene therapies having extremely high prices, limiting widespread adoption.^[134,135]^ In addition, current gene therapy production relies on specialized bioprocessing techniques that are difficult to scale, limiting availability, and the variability in gene expression, vector integration, and dosing makes standardization possible.^[136,137]^

### 4.5. Cytotherapy

#### 4.5.1. Stem cell therapy

Mesenchymal stem cells (MSCs) have garnered considerable attention in KOA research owing to their unique biological properties.^[127]^ These cells possess the ability to self-renew and differentiate into various cells, including chondrocytes, which are crucial for cartilage regeneration.^[138,139]^ Additionally, MSCs exhibit immunomodulatory effects that can help mitigate the inflammatory processes associated with KOA pathogenesis. Their multilineage differentiation potential, particularly their capacity to differentiate into chondrocytes, makes them attractive candidates for cell-based therapies aimed at repairing damaged cartilage in KOA joints. Clinical trials investigating the efficacy of autologous MSC therapy in KOA have primarily yielded limited evidence regarding pain relief and functional improvement in patients (NCT01183728, NCT03869229, NCT05081921, NCT05783154). Furthermore, these trials have not provided convincing evidence of significant changes in cartilage thickness as assessed by magnetic resonance imaging.^[114–116,140]^ Despite the promising regenerative potential of MSCs, the outcomes of these clinical studies suggest that further research is necessary to optimize treatment protocols and better understand the mechanisms underlying MSC-based therapies for KOA.^[117]^

Exosomes are a subtype of extracellular vesicles secreted by cells that facilitate intercellular communication.^[141]^ These nanosized extracellular vesicles carry diverse cargoes of bioactive molecules, including proteins, lipids, and nucleic acids, which can modulate cellular processes involved in tissue repair and regeneration. Mesenchymal stem cell-derived exosomes (MSC-Exos) have garnered considerable attention in biomedical research owing to their potential therapeutic applications in treating KOA.^[142]^ Studies have demonstrated the ability of MSC-Exos to promote chondrogenesis and autophagy, inhibit inflammation, and enhance extracellular matrix synthesis in preclinical models of cartilage damage.^[143–145]^ MSC-Exos can also be developed as delivery vehicles loading active compounds for targeted therapy under KOA conditions.^[146]^ Recently, a phase I study was performed to assess the safety profile of MSC-Exos administered via intra-articular injection in the knee joints of patients diagnosed with mild to moderate symptomatic osteoarthritis (NCT05060107). Further research is warranted to elucidate the mechanisms of action and optimize the therapeutic efficacy of MSC-Exos in clinical settings.

#### 4.5.2. Platelet therapy

Emerging autologous cellular therapies have shown potential as adjunctive components in various regenerative medicine treatment protocols.^[147]^ Platelet therapy based on platelet-rich plasma (PRP) is characterized as a component of autologous blood with an elevated platelet concentration compared to baseline levels.^[148]^ PRP therapies have been used for various indications for over 3 decades, garnering considerable attention for their potential in regenerative medicine.^[149]^ Platelet growth factors facilitate 3 phases of the wound healing and repair cascade: inflammation, proliferation, and remodeling. However, recommendations derived from in vitro and animal research often yield divergent clinical outcomes because the challenge of translating nonclinical study findings and clinical treatment protocols.^[150]^ While intra-articular injections of PRP may be advisable for patients with KOA, it is important to note that this approach lacks FDA approval.^[151]^

While cell therapy holds great promise for treating various diseases, potential risks include immune rejection, tumorigenicity (e.g., uncontrolled proliferation of stem cells), and complications from immune-modulating therapies. Challenges related to cost, scalability, and standardization hinder its widespread adoption.^[152,153]^ Addressing these issues through technological advancements, automation in manufacturing, and improved regulatory frameworks is essential for making cell therapies more accessible and feasible on a global scale.

## 5. Conclusions and future prospects

Preclinical studies and genetic analyses in KOA research have identified numerous novel therapeutic targets through comprehensive investigations into disease mechanisms and genetic factors, offering potential avenues for the development of innovative targeted therapy strategies. Some targeted drugs have progressed to phase II and III clinical trials, where they have shown promising results in terms of efficacy and safety profiles. Nanocarrier-based therapy offers precise control over drug delivery, allowing for enhanced therapeutic efficacy, sustained drug release and reduced systemic side effects. Depending on the therapeutic goals, these systems can target specific tissues within the joint, such as cartilage, synovium, or cells residing within these tissues. By precisely delivering therapeutic agents to the desired sites of action, nano-scale drug delivery systems hold great promise for improving KOA treatment outcomes. Their ability to selectively target affected tissues while minimizing off-target effects represents a significant advancement in the field of KOA therapy. The majority of targeted therapy strategies used clinically consist of small-molecule inhibitors and mAbs, which are continuously under investigation for their potential applications in KOA treatment. Research in this area is ongoing with a focus on developing disease-modifying osteoarthritis drug (DMOADs). Over the next decade, substantial advancements have been made in OA therapeutics, driven by ongoing research and development efforts focused on small-molecule inhibitors and mAbs. Currently, gene therapy and cytotherapy for KOA are gaining significant attentions with advancements made in addressing the challenge of sustaining transgene expression in joints and supplying cartilage regeneration therapy over the long term. However, there is a lack of consensus regarding the optimal selection of transgenes and promoters for effective treatment. There is a growing interest in utilizing multiple therapeutic gene products as they may offer enhanced efficacy. Additionally, exploring combination therapeutic approaches that integrate gene therapy with other medical or surgical interventions warrants further investigation. Despite significant progress in the field of MSC-based regenerative medicine, several challenges persist, indicating that there is still a long road ahead to effectively and economically repair articular cartilage defects and osteochondral interface. To achieve efficient osteogenesis and chondrogenesis, innovative strategies and technologies are required to enhance the potential of cytotherapies for KOA. In conclusion, all advancements in targeted therapy underscore the importance of translational research in elucidating new therapeutic options for KOA and provide hope for improved management and outcomes for patients with this debilitating condition.

## Author contributions

**Conceptualization:** Qi Tang.

**Formal analysis:** Juan-Hong Liang.

**Investigation:** Feng-Lan Huang, Zhen-Yan Ye.

**Methodology:** Feng-Lan Huang.

**Visualization:** Zhen-Yan Ye.

**Writing –original draft:** Li Chen.

**Writing – review & editing:** Zhi-Kai Zhao, Juan-Hong Liang.
